# Wheat *WCBP1* encodes a putative copper-binding protein involved in stripe rust resistance and inhibition of leaf senescence

**DOI:** 10.1186/s12870-015-0612-4

**Published:** 2015-10-06

**Authors:** Xin Li, Taiguo Liu, Wanquan Chen, Shengfu Zhong, Huaiyu Zhang, Zongxiang Tang, Zhijian Chang, Ling Wang, Min Zhang, Liqin Li, Hefei Rao, Zhenglong Ren, Peigao Luo

**Affiliations:** State Key Laboratory for Biology of Plant Diseases and Insect Pests, Institute of Plant Protection, Chinese Academy of Agricultural Sciences (CAAS), Beijing, 100193 China; Provincial Key Laboratory of Plant Breeding and Genetics, Sichuan Agricultural University, Chengdu, Sichuan 611130 China; Institute of Crop Genetics, Shanxi Academy of Agricultural Science, Taiyuan, Shanxi 030031 China

**Keywords:** Quantitative reverse transcription PCR, *Puccinia striiformis*, *Triticum aestivum*, Suppression subtractive hybridization, Photosynthesis

## Abstract

**Background:**

Stripe rust, a highly destructive foliar disease of wheat (*Triticum aestivum*), causes severe losses, which may be accompanied by reduced photosynthetic activity and accelerated leaf senescence.

**Methods:**

We used suppression subtractive hybridization (SSH) to examine the mechanisms of resistance in the resistant wheat line L693 (Reg. No. GP-972, PI 672538), which was derived from a lineage that includes a wide cross between common and *Thinopyrum intermedium*. Sequencing of an SSH cDNA library identified 112 expressed sequence tags.

**Results:**

*In silico* mapping placed one of these tags [GenBank: JK972238] on chromosome 1A. Primers based on [GenBank: JK972238] amplified a polymorphic band, which co-segregated with *YrL693*. We cloned a candidate gene encoding wheat copper-binding protein (WCBP1) by amplifying the polymorphic region, and we mapped *WCBP1* to a 0.64 cM genetic interval. *Brachypodium*, rice, and sorghum have genes and genomic regions syntenic to this region.

**Discussion:**

Sequence analysis suggested that the resistant *WCBP1* allele might have resulted from a deletion of 36-bp sequence of the wheat genomic sequence, rather than direct transfer from *Th. intermedium*. qRT-PCR confirmed that *WCBP1* expression changes in response to pathogen infection.

**Conclusions:**

The unique chromosomal location and expression mode of *WCBP1* suggested that *WCBP1* is the putative candidate gene of *YrL693*, which was involved in leaf senescence and photosynthesis related to plant responses to stripe rust infection during the grain-filling stage.

**Electronic supplementary material:**

The online version of this article (doi:10.1186/s12870-015-0612-4) contains supplementary material, which is available to authorized users.

## Background

Stripe rust, caused by the fungus *Puccinia striiformis* f. sp. *tritici* (*Pst*), is one of the most destructive diseases affecting wheat (*Triticum aestivum* L.) in many regions of the world. In China, stripe rust infects millions of hectares of wheat annually [1], causing large yield losses over the past two decades due to the emergence of new virulent races in the fungus population [2, 3].

As a foliar disease, stripe rust has a considerable effect on grain yield, depending on the disease response [4] and the host growth stage at which *Pst* infection is initiated [5]. Stripe rust epidemics can begin at any growth stage in wheat [6, 7], and therefore, they have a marked effect on plant vigor in addition to grain yield [8]. Severe stripe rust infection also alters the distribution of assimilates among the various organs of the plant [9, 10].

Stripe rust infection is closely linked to both leaf photosynthesis and senescence, and many of the expressed sequence tags (ESTs) induced by *Puccinia striiformis* are related to those two processes [11–14]. A recent study in wheat showed that the senescence-associated gene (SAG) *TaSAG120* plays an important role in the defense against stripe rust [15]. In addition, the multi-gene pathogen resistance factor ‘*Lr34*/*Yr18*/*Pm38*’ involves leaf senescence [16]. These findings imply that senescence-and photosynthesis-related processes might be part of the resistance response to stripe rust infection.

Wheat line L693 (Reg. No. GP-972, PI 672538) is from F_6:7_ families of a cross between MY11 and YU25, the latter being a derivative of a wide cross involving *Thinopyrum intermedium* [17]. Genetic analysis demonstrated that stripe rust resistance observed in L693 is conferred by a single dominant gene, *YrL693*, which was mapped to chromosome 1B by linkage analysis with simple sequence repeat (SSR) markers and Chinese Spring nulli-tetrasomic lines [18, 19]. Although the pedigree of L693 includes *Th. intermedium*, the question of whether *YrL693* is derived directly from *Th. intermedium* remains unanswered.

The objectives of the present study were to assess whether resistant plants maintained the normal timing of leaf senescence during grain filling and to examine the physiological effects of any leaf senescence traits that were affected by infection. We also explored the molecular mechanism of resistance to stripe rust in wheat and attempted to determine the origin of *YrL693* by high-density mapping and comparative genomic analysis. We demonstrated that it is possible to map resistance genes using the identified ESTs between isogenic lines after pathogen infection, and we outline a putative novel genetic mechanism of resistance conferred by this gene.

## Results

### Identification of unique ESTs induced by *Pst* infection

**Fig. 1:**
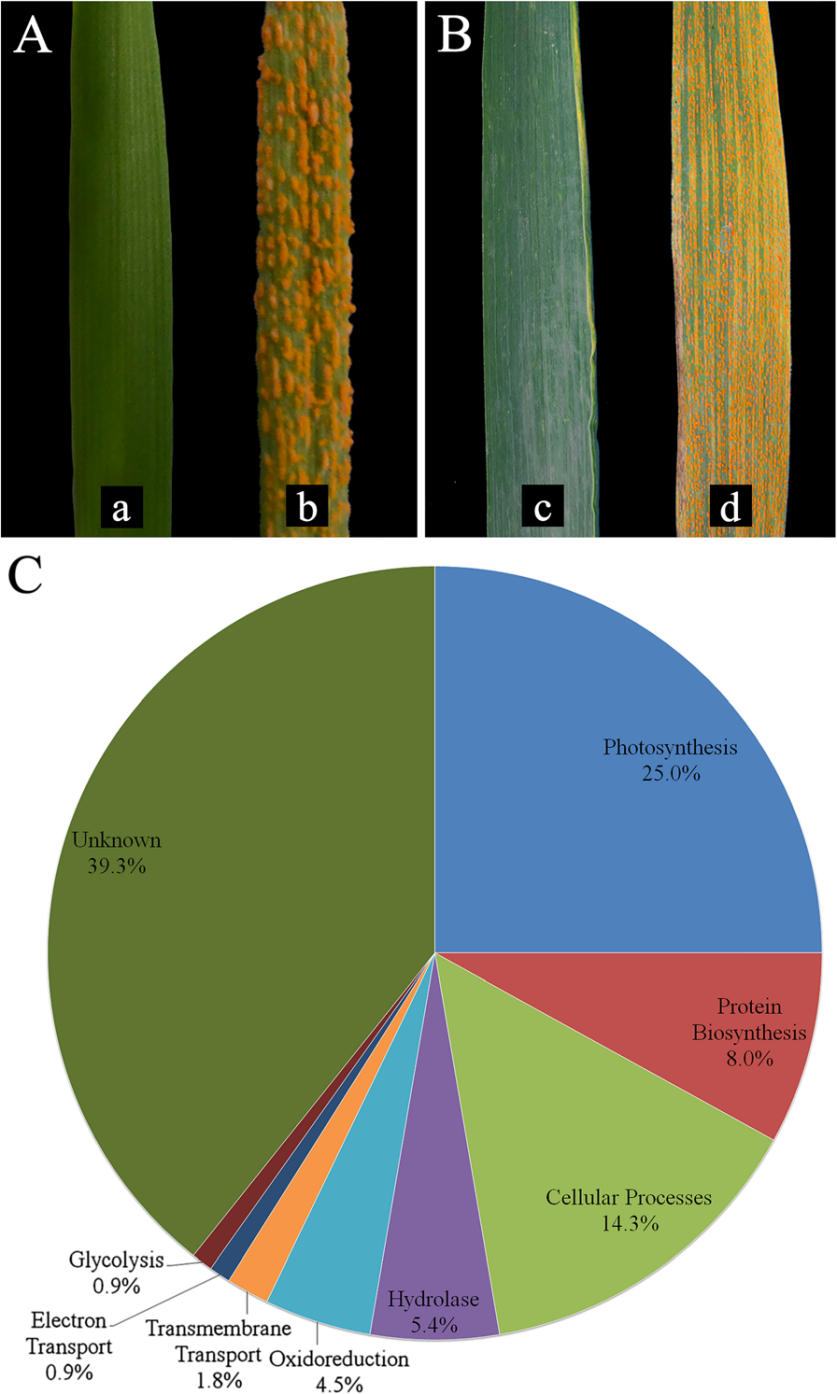
Seedling (**a**) and adult (**b**) responses to *a*) L693, *b*) L661, *c*) L693, *d*) L661. **c** Functional classes of differentially expressed sequences induced by *Pst* inoculation

L693 was highly resistant and L661 highly susceptible to *Pst* race CYR32 (Fig. 1a). The response tests performed at the adult (Fig. 1b) and seedling stages (Fig. 1a) showed similar results. Random sequencing of bacterial colonies from the suppression subtractive hybridization (SSH) library identified 112 ESTs, with an average length of 178 bp, which were submitted to NCBI under the accession numbers [GenBank: JK972179] though [GenBank: JK972287] and [GenBank: JK974072] through [GenBank: JK974074] (Additional file 2: Table S1). Of these, 87 unique ESTs (uniESTs) with an average length of 197 bp (Table 1), including 21 contigs with an average length of 226 bp and 66 singletons with an average length of 188 bp, were found. Most of the uniESTs were between 50 bp and 300 bp in length (Additional file 1: Figure S2) and were further identified through clustering and assembly of putative full-length cDNAs based on the BLAST analysis option in NCBI GenBank (Additional file 2: Table S2).

**Table 1:** ESTs in the SSH library prepared from combined leaf samples obtained from wheat seedlings

	Number obtained	Average length (bp)
ESTs	112	178
Singletons	66	188
Contigs	21	226
UniESTs	87	197

Both L693 and L661 were sampled 24, 48 and 96 h after inoculation with *Pst* CYR32. cDNA prepared from L693 leaves infected with *Pst* CYR32 was used as the tester, and cDNA prepared from L661 leaves infected with *Pst* CYR32 was used as the driver

Functional categorization of the ESTs, as well as gene network analysis, predicted that *Pst* infection in L693 induces differential regulation of leaf senescence-associated-genes (SAGs).

Of the 112 sequences, 68 showed high sequence similarity to known or annotated genes from cDNA libraries from wheat, rice, barley, pea and *Arabidopsis thaliana* (Fig. 1c, Additional file 2: Table S2). Of the genes that were upregulated in L693, 25 % were involved in photosynthesis-like and leaf senescence-like pathways, 14 % in cellular process pathways, 8 % in protein biosynthesis pathways, 5 % in the hydrolase pathway, 5 % in the oxidoreduction pathway, 2 % in the transmembrane transport pathway, 1 % in the electron transport pathway, and 1 % in glycolysis. The remaining 44 (39 %) of upregulated sequences had unspecified or unknown functions, 14 of which closely matched the sequences of putative senescence-associated genes (Fig. 1c and Additional file 2: Table S2).

### Polymorphisms between ESTs and linkage analysis of stripe rust resistance

**Fig. 2:**
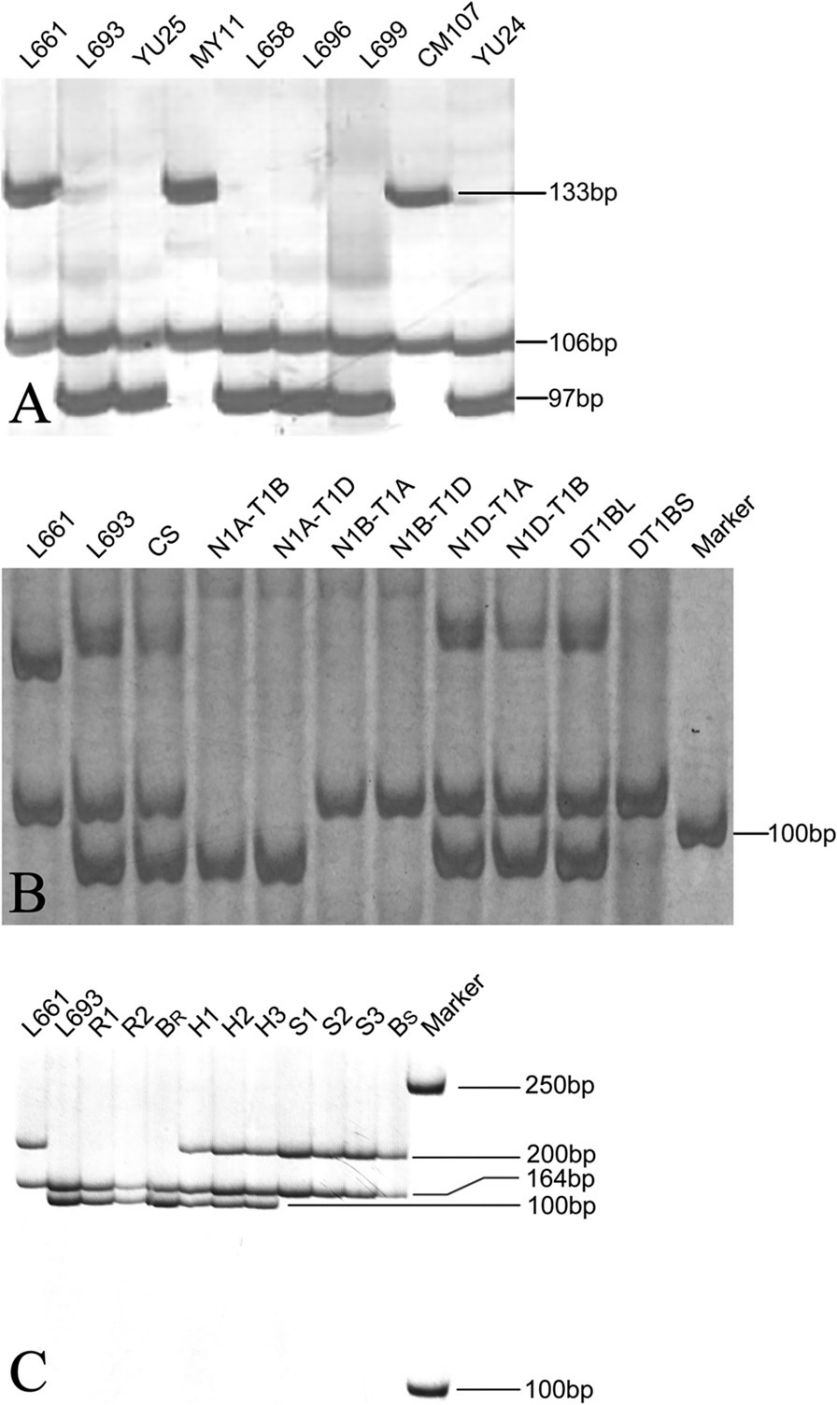
Identification of polymorphism between stripe rust-resistant and stripe rust-susceptible genotypes. The Ls36 primer designed from an EST sequence [GenBank: JK972238] produced polymorphic amplicons among the various genotypes. **a** a 133 bp amplicon was amplified in the susceptible L661 line and its susceptible parents MY11 and CM107. A 97 bp amplicon was amplified in resistant L693 line, resistant sister lines L658, L696, and L699, and their resistant parent, YU25. A 97 bp amplicon was also amplified from YU24, one of the resistant sister lines of YU25. A 106 bp fragment was amplified in all genotypes. **b** chromosomal localizations of the amplicons that were polymorphic between L693 and L661, with CS nulli-tetrasomic lines of homoeologous group 1 and ditelosomic lines of wheat chromosome 1B. PCR was performed to map the gene using genomic DNA from the CS and various aneuploids. PCR products were resolved on 6 % polyacrylamide gels. No PCR product was generated from nullisomic 1B (N1BT1A and N1BT1D) or the ditelosomic 1BS (DT1BS) lines. **c** Silver-stained polyacrylamide gels showing polymorphic markers generated using the LSc18 primer linked to the stripe rust resistance gene in L693. L661, susceptible parent; L693, resistant parent; R1 and R2, resistant F_2_ individuals; B_R_, the resistant F_2_ DNA pool; H1, H2 and H3, resistant F_2_ individuals; S1, S2 and S3, susceptible F_2_ individuals; B_S_, the susceptible F_2_ DNA pool. L661, S1, S2, S3 and B_s_ showed amplification of 173 bp and 200 bp fragments; L693, R1, R2 and B_R_ showed amplification of 164 bp and 173 bp fragments; H1, H2 and H3 showed amplification of all three fragments, indicating heterozygosity

Out of 62 primers designed, one primer pair (LS36) (Additional file 2: Table S3) produced polymorphic amplicons between L693 and L661 as well as between the resistant parent YU25 and the susceptible parent MY11 (Fig. 2a). The design of the LS36 primers was from the sequence of [GenBank: JK972238], which we mapped to wheat chromosome 1A by *in silico* mapping. The L693, L661, YU25 and MY11 lines produced two amplicons, one 106 bp amplicon, which was the same among all genotypes, and a second amplicon that was polymorphic between L693 and L661 and between YU25 and MY11. In L661 and susceptible parents MY11 and CM107, this amplicon was 133 bp, and in the resistant lines L693, L658, L696 and L699, as well as YU25 and sister line YU24, the amplicon was 97 bp. Amplification of genomic DNA from the nullisomic-tetrasomic and ditelosomic lines (Fig. 2b) revealed that the amplicon common among all lines and CS was absent in the nullisomic 1A lines (N1AT1B and N1AT1D).

The polymorphic 97 bp amplicon was located on chromosome arm 1BL, as no PCR products were generated from the nullisomic 1B lines (N1BT1A and N1BT1D) or the ditelosomic 1BS line (DT1BS). Sequencing of the polymorphic amplicon found that there was an extra repeat of 36 bp sequence in the susceptible allele compared with the resistant allele. Sequence similarity searches with the polymorphic amplicon found a contig (3837062) with high sequence similarity located on wheat chromosome 1B.

We designed four pairs of primers for linkage analysis based on differences in the number of copies of the 36 bp sequence (Additional file 2: Table S4). Analysis using the LSc18 primer pair (Fig. 2c) detected a 100 bp product closely linked to the *YrL693* resistance gene in L693. Analysis of 523 F_2:3_ lines with additional primer pairs (Additional file 2: Table S5) further demonstrated a co-dominant marker on chromosome 1B co-segregating with *YrL693*.

### Fine mapping and comparative genomic analysis of *WCBP1* indicated that it co-segregated with *YrL693*

**Fig. 3:**
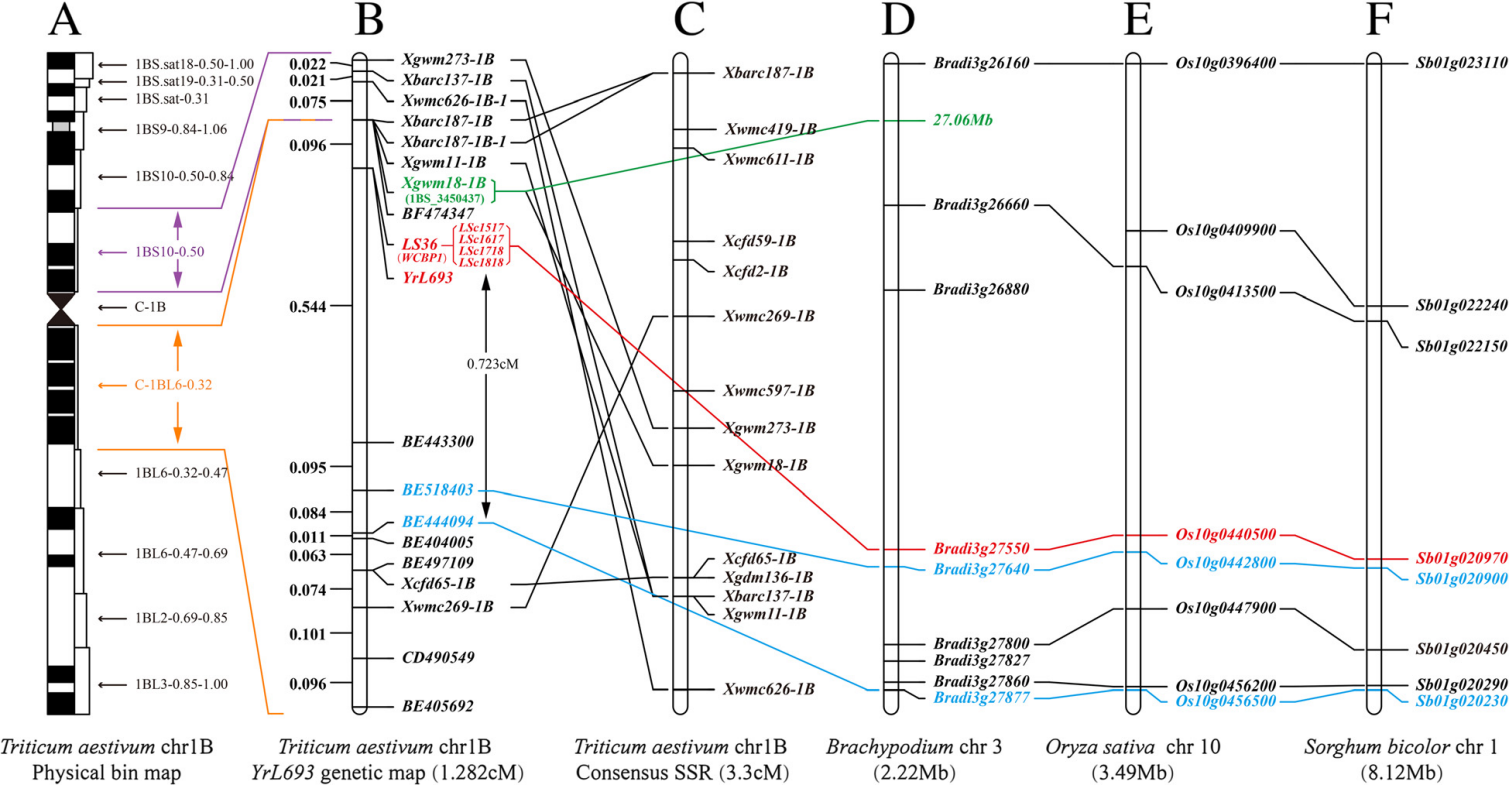
Genetic and comparative genomic linkage map of the candidate gene *WCBP1* and *YrL693*. **a** Physical bin map of *WCPB1*; *WCBP1* was mapped to the bin C-1BL6-0.32. **b** Genetic map of *WCBP1* with a total length of 1.28 cM on wheat chromosome 1B. The genetic distance in cM is shown on the left and genetic markers are shown on the right. **c** The consensus SSR map for a total of 3.3 cM of wheat chromosome 1B. **d**, **e**, **f**, Orthologous regions of *WCBP1* in *Brachypodium distachyon* chromosome 3, and *Oryza sativa japonica* chromosome 10, and *Sorghum bicolor* chromosome 1, respectively

Genomic *in situ* hybridization (GISH) failed to detect an alien chromosome segment from *Th. intermedium* in L693 (Additional file 1: Figure S6; Additional file 3: Methods S1). A high-density integrated genetic map was constructed (Fig. 3). The linkage map consisted of nine SSR markers and eight EST-STS markers (Additional file 2: Table S8). The *WCBP1* locus fell within a genetic interval of 0.64 cM. *WCBP1* co-segregated with the *YrL693* locus. Two flanking EST-STS markers, *BF474347* and *BE443300*, were linked to the *WCPB1* locus at 0.096 and 0.544 cM, respectively, and mapped to the chromosome 1B bin C–1BL-6-0.32. Therefore, *WCBP1* is located in the same chromosomal region as *YrL693*. The contigs of the two SSR markers *Xwmc269-1B* and *Xcfd65-1B* on one side of *WCBP1* mapped to wheat chromosomal arm 1BL, whereas those of the seven other SSR markers on the other side of *WCBP1* mapped to 1BS according to the published SSR linkage map [20].

**Fig. 4:**
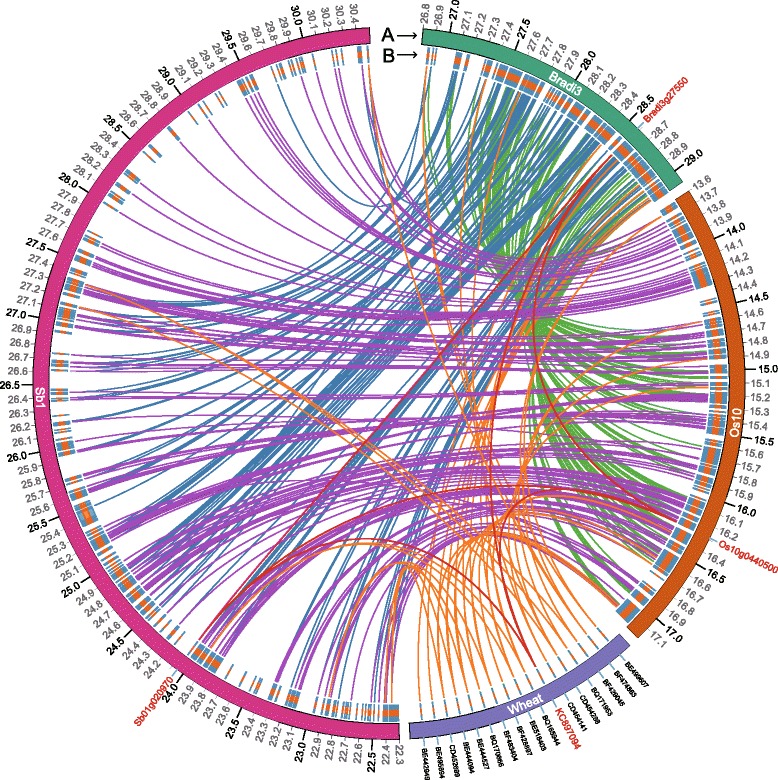
Comparative genomic mapping of ESTs in the *WCBP1* (*red*) chromosomal region of wheat chromosome 1B (*purple*) with *Brachypodium distachyon* chromosome 3 (*green*), *Oryza sativa japonica* chromosome 10 (*orange*), and *Sorghum bicolor* chromosome 1 (*red*). The gene order in all species is clockwise

Orthologs of wheat ESTs BE518403 and BE444094 were detected on *B. distachyon* chromosome 3, rice chromosome 10, and sorghum chromosome 1. Comparative genomic analysis established the collinearity of *WCBP1* genomic regions with a 2.22 Mb (*Bradi3g26160*–*Bradi3g27877*) region on *B. distachyon* chromosome 3, a 3.49 Mb region (*Os10g0396400*–*Os10g0456500*) on rice chromosome 10, and a 8.12 Mb region (*Sbo1g020230*–*Sb01g023110*) on sorghum chromosome 1, regions that carry 112, 260, and 297 genes, respectively. The gene order was highly conserved among wheat, *B. distachyon*, and rice (Fig. 4), but showed an intra-chromosomal inversion in the 8.12 Mb chromosome region of sorghum. We also found an orthologous genomic region (from 27,059,150 to 27,060,251) of wheat contig 1BS-3450437 carrying the SSR marker *Xgwm18-1B* at 27.06 Mb on *B. distachyon* chromosome 3 (E-value = 5.34E^−34^), but there was no corresponding ortholog in either rice or sorghum (Fig. 3). An ortholog of *WCBP1* was found on *Bradi3g27550* (E-value ≈ 0) at 28 Mb on *B. distachyon* chromosome 3, on *Os10g0440500* (E-value = 5.34E^−168^) at 16 Mb on rice chromosome 10, and on *Sb01g020970* (E-value = 1.14E^−130^) at 24.05 Mb on sorghum chromosome 1 (Figs. 3 and 4).

### PCR-based cloning and functional analysis of the candidate stripe rust resistance gene

We obtained a candidate resistance gene by sequencing PCR products amplified by seven primers (Additional file 2: Table S6) from genomic DNA and then comparing the amplified sequence with that of the contig (3837062). This gene was 1,398 bp in the resistant lines L693 and YU25 but 1,434 bp in the susceptible lines L661 and MY11 (Additional file 1: Figure S4). The genomic sequence of the candidate resistance gene isolated from L693 and YU25 included four exons (154, 329, 79, and 479 bp) and three introns (112, 126, and 119 bp). The fourth exon contained a single repeat of the 36 bp sequence in the resistant L693 line and its parent YU25 as well as two repeats in the susceptible L661 line and its parent MY11. We also identified and verified a single nucleotide polymorphism (SNP) in the first exon, with an adenine (A) in the resistant lines and a cytosine (C) in susceptible lines L661 and MY11 (Additional file 1: Figure S5).

**Fig. 5:**
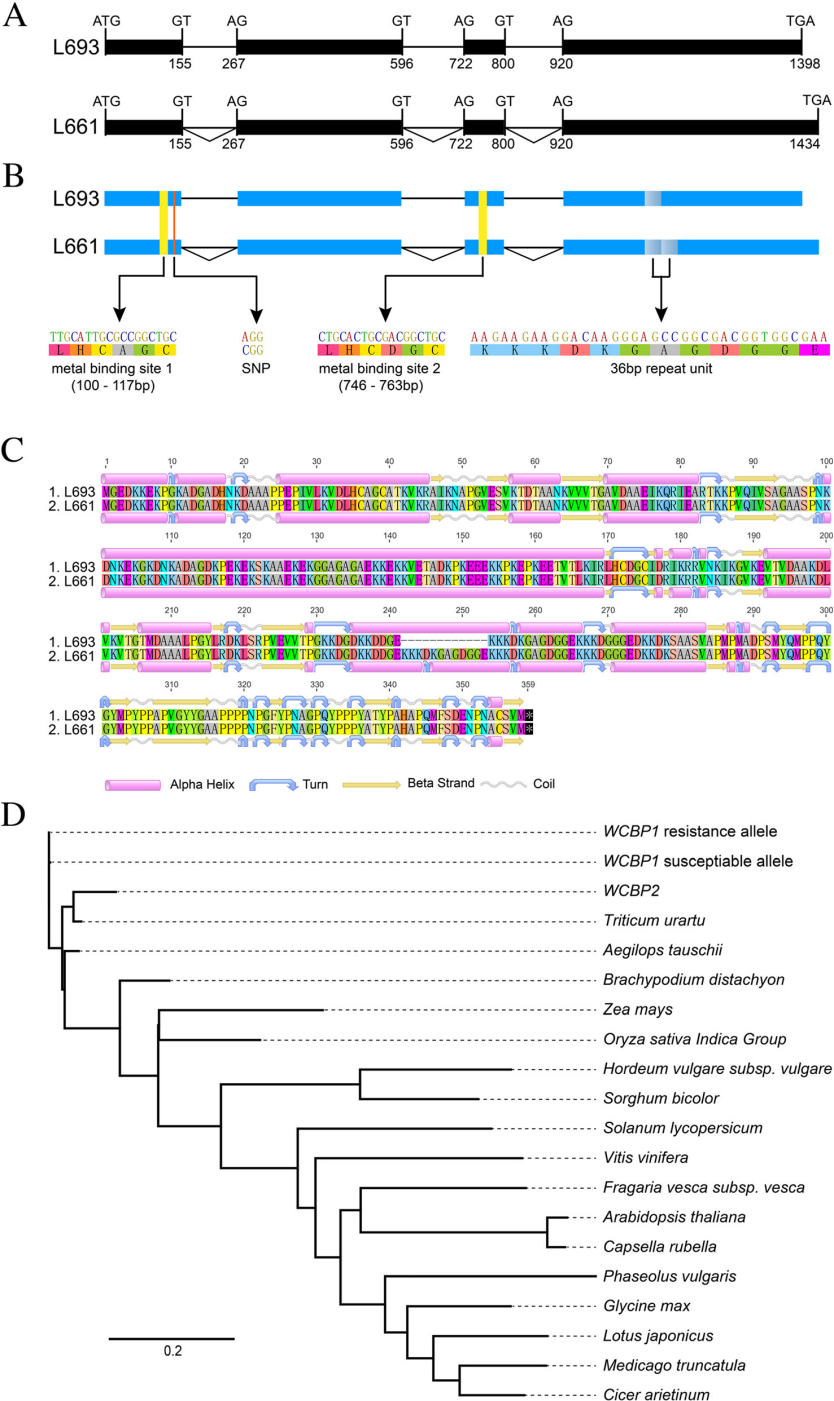
The *WCBP1* gene, its putative protein structure, and its phylogenetic tree. **a** The predicted mRNA structure of *WCBP1* is shown above the genomic sequence, including introns (thin lines) and exons (thick lines). ATG represents the methionine start codon, and TGA represents the stop codon. **b** Coding regions of two heavy metal copper-binding domains in exons 1 and 3. The major differences between the resistance-and susceptibility-associated *WCBP1* alleles are a SNP in exon 1 and a 36 bp deletion in exon 4. **c** Alignment of the amino acid sequences and secondary structures of the proteins putatively encoded by the resistance-and susceptibility-associated alleles of *WCBP1*. **d** Phylogenetic tree constructed using the neighbor-joining algorithm in MEGA 5.05 following WCBP1 protein sequence alignment using the CLUSTALW program. Accession numbers for the other heavy metal copper-binding proteins were as follows: EMS53947 (*Triticum urartu*) (11.37 %), EMT15307 (*Aegilops tauschii*) (11.37 %), XP_003573974 (*Brachypodium distachyon*) (11.37 %), EAY78671 (*Oryza sativa* indica group) (3.72%), XP_002465840 (*Sorghum bicolor*) (6.65 %), AFK36536 (*Medicago truncatula*) (4.72 %), XP_004497534 (*Cicer arietinum*) (4.72%), XP_004955283 (*Setaria italica*) (4.58 %), BAK03814 (*Hordeum vulgare* subsp. *vulgare*) (4.15 %), XP_004304460 (*Fragaria vesca* subsp*. vesca*) (4.01%), DAA49855 (*Zea mays*) (3.93 %), NP_195958 (*Arabidopsis thaliana*) (3.79 %), EOA20845 (*Capsella rubella*) (3.79 %), XP_004246628 (*Solanum lycopersicum*) (3.72 %), XP_003542527 (*Glycine max*) (3.72 %), AFK35929 (*Lotus japonicus*) (3.72 %), EAY78671 (*Oryza sativa* indica group) (3.72%), XP_002276537 (*Vitis vinifera*) (3.72%) and AFW90521 (*Phaseolus vulgaris*) (3.29 %)

The open reading frame (ORF) (Fig. 5a) of the candidate resistance gene encodes a putative wheat copper binding protein 1 (*WCBP1*) predicted to contain 346 amino acids in resistant lines (AGS38338) and 358 amino acids in susceptible lines. The SNP in the first exon (Additional file 1: Figure S5) does not change the deduced amino acid sequence. The 36 bp repeated sequence in the fourth exon (Fig. 5b and Additional file 1: Figure S3) caused an addition of 12 amino acids (K K K D K G A G D G G E) in the susceptible line (Fig. 5b) at amino acids 242–253. The deduced amino acid sequence forms a protein containing two predicted copper-binding domains, at amino acids 34–39 (L H C A G C, exon 1), and at amino acids 170–175 (L H C D G C, exon 3) (Fig. 5b). The full-length, deduced WCBP1 protein shows sequence similarity with known heavy metal-binding proteins from diverse species. Phylogenetic analysis indicated that WCBP1 shared the highest similarity with the EMS53947 protein (Fig. 5d), but they did not cluster together.

Sequence analysis identified a highly similar gene, *WCBP2*, on chromosome 1A. This gene had the same DNA sequence in L693 and L661. *WCBP1* and *WCBP2* show 91.6 % DNA sequence similarity. Moreover, no polymorphism in the length of fragments was detected in genomic DNA of resistant and susceptible varieties.

### *WCBP1* and *WCBP2* are differentially regulated in association with resistance

**Fig. 6:**
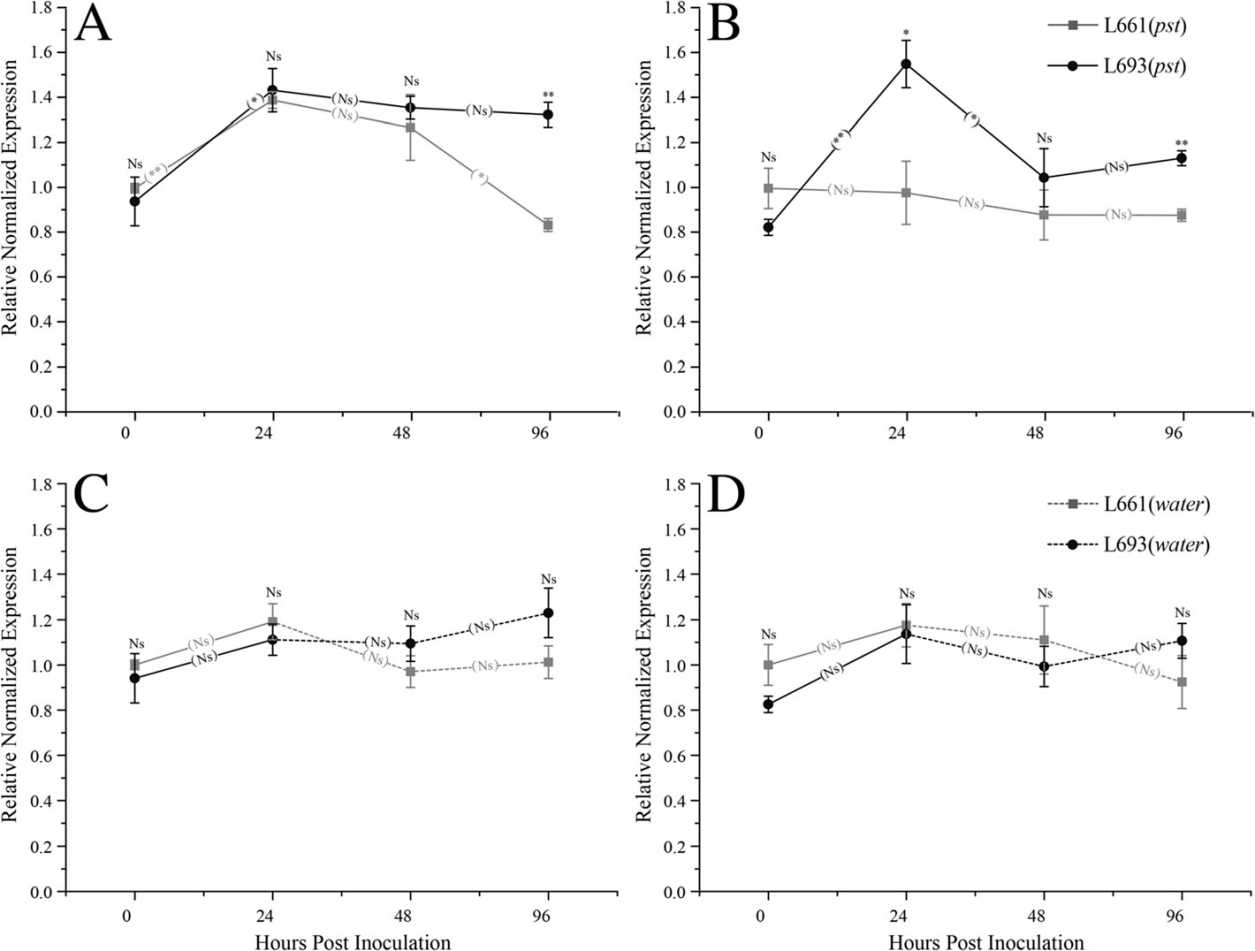
qRT-PCR expression analysis of *WCBP1* and *WCBP2*. Amplification of *WCBP1* (**a**) and *WCBP2* (**b**) transcripts following *Pst* infection. C, Amplification of *WCBP1* (**c**) and *WCBP2* (**d**) transcripts following mock inoculation with water. The experiment was performed using three biological replicates with three technical replicates per biological sample. Relative expression was calculated using *GAPDH* as a reference gene to infer steady-state mRNA levels, and relative expression was normalized by setting the control sample from L661 at 0 h to 1. Bars represent the standard error, and the significance was determined using independent sample t-tests. Asterisks represent significant differences as follows: **P ≤0.01, *P ≤0.05; Ns, no significant difference. The raised asterisks or the Ns designation represent differences in gene expression between L693 and L661 at each time point. Asterisks or the Ns designation in the trend line represent the differences in gene expression between two adjacent time points in the same genotype

To examine possible involvement of *WCBP1* in defense against stripe rust, we measured its transcript levels at different time points following pathogen inoculation (Additional file 2: Table S7). *WCBP1* [NCBI GenBank: KC897094] was upregulated in leaves following *Pst* inoculation of both the resistant and the susceptible parent. Transcript abundance of *WCBP1* was significantly higher (*P* <0.05) in leaves from L661 and L693 at 24 h post inoculation than prior to inoculation (Fig. 6). However, at later time points, the *WCBP1* transcript abundance decreased in L661 but remained higher in L693. At 96 h post-inoculation, the *WCBP1* transcript abundance was significantly lower in L661 compared to L693 (*P* <0.01, Fig. 6a).

We also measured the transcript levels of *WCBP2*. Prior to inoculation, L693 and L661 showed similar *WCBP2* transcript levels, but after inoculation, *WCBP2* was upregulated only in the resistant L693 line. At 24 h post-inoculation, *WCBP2* transcript abundance in L693 increased significantly compared with its level prior to inoculation (*P* <0.01), and *WCBP2* transcript levels were significantly higher (*P* <0.01) in L693 than in L661 at both 24 h and 96 h post-inoculation (Fig. 6b). In addition, we found that mock inoculation with water had no significant effects on the transcript level of either *WCBP1* or *WCBP2* in L693 or L661 at any time point (Fig. 6c and d).

### The effects of resistance on photosynthesis, chlorophyll fluorescence dynamics, and yield

**Fig. 7:**
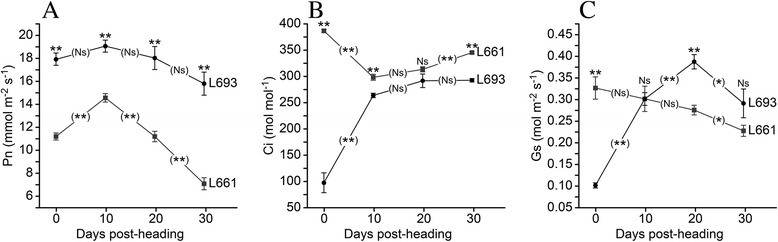
Photosynthetic parameters used to assess the different responses of L693 and L661 to *Pst* inoculation. **a**, Net photosynthetic rate (*P*_*n*_); **b**, intercellular CO_2_ concentration (*C*_*i*_); **c**, stomatal conductance (*G*_*s*_). The parameters were measured once every 10 days after heading in the field, using a Li-Cor 6400. The disease had fully developed at heading, and 10 plants per genotype were used for these measurements. Bars represent the standard error, and significance was determined using an independent sample *t*-test. Asterisks represent significant differences as follows: **P ≤0.01 and *P ≤0.05, and Ns indicates no significant difference. Raised asterisks or the Ns label represent the differences in gene expression between L693 and L661 at each time point. Asterisks or the Ns label on the trend line represent differences in gene expression between two adjacent time points in the same genotype

Although the net photosynthetic rates (*P*_*n*_) at all time points were significantly higher (*P* <0.01) in L693 than L661, these genotypes showed similar trends (Fig. 7). Notably, there were no significant differences (*P* >0.05) between any two consecutive measurements in L693, whereas changes in L661 were highly significant (*P* <0.01) (Fig. 7a). At the heading stage, the stomatal conductance (*G*_*s*_) in L693 was significantly lower than that in L661 (Fig. 7b). The *G*_*s*_ of L693 increased sharply during the early stages and decreased toward the end of the experiment, while the values obtained for L661 declined sharply during the early stages of the experiment and then increased. The intercellular CO_2_ concentrations (*C*_*i*_) observed in L693 were significantly lower (*P* <0.01) than those in L661 at all time points, except at 20 days after heading (Fig. 7c). In L693, there was a notable increase in *C*_*i*_, whereas this value decreased sharply between heading and 10 days after heading in L661.

**Fig. 8:**
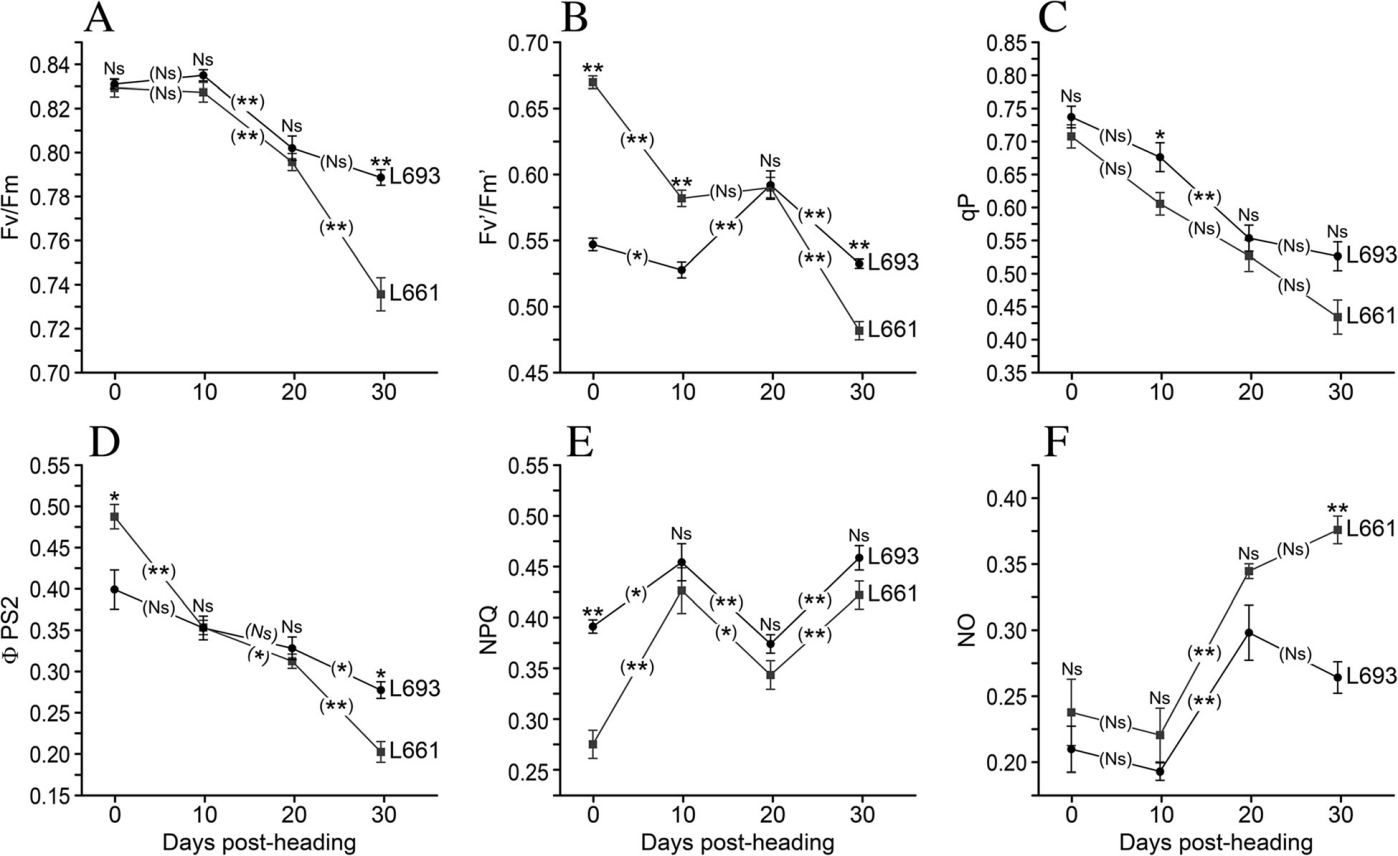
Chlorophyll fluorescence-related parameters used to determine differences in photosystem II (PSII) activity between the resistant line L693 and the susceptible line L661 following *Pst* inoculation and after heading. **a**, Values for the maximal photochemical efficiency of PSII (*F*_*v*_ */F*_*m*_); **b**, the efficiency of excitation capture by the open PSII reaction centers (*F’*_*v*_ */F’*_*m*_); **c**, the photochemical quenching coefficient (*qP*); **d**, quantum yield of photochemical energy conversion in PSII (*ΦPSII*); **e**, quantum yield of regulated non-photochemical energy loss in PSII (*NPQ*); and **f**, quantum yield of non-regulated non-photochemical energy loss in PSII (*NO*) in wheat flag leaves. The parameters *ΦPSII*, *NPQ* and *NO* were derived from averages of related parameters according to a previously described method [36]. The disease had fully developed at heading, and 10 plants per genotype were used for the measurements. Bars represent the standard error, and significance was determined using independent sample t-tests. Asterisks represent significant differences as follows: **P ≤0.01, *P ≤0.05, and Ns for no significant difference. Raised asterisks or NS represent the differences in gene expression between L693 and L661 at each time point. The asterisks or the Ns label on the trend line represent differences in gene expression between two adjacent time points in the same genotype

Although the changes in the maximal photochemical efficiency of PSІІ in the dark-adapted leaves (*F*_*v*_/*F*_*m*_) were similar between L693 and L661, the *F*_*v*_/*F*_*m*_ value obtained for L693 was significantly higher than that obtained for L661 at 30 days after heading. A significant decrease in *F*_*v*_/*F*_*m*_ occurred in L661 between 20 days and 30 days after heading, whereas only a slight decrease occurred in this timeframe in L693 (Fig. 8a). The efficiencies of excitation capture by the open PSII reaction centers (*F*_*v*_*‘*/ *F*_*m*_*’*) observed in L693 were significantly lower than, similar to, and higher than those recorded in L693 prior to 20 days, at 20 days and at 30 days after heading, respectively (Fig. 8b). The photochemical quenching coefficient (*q*P) was similar in L693 and L661. Although the *q*P values obtained in L693 were greater than those in L661, the differences were not significant at the *P* = 0.05 level (Fig. 8c). The changes in the quantum yield of photochemical energy conversion in PSII (*ΦPSII*) (Fig. 8d), the quantum yield of regulated non-photochemical energy loss in PSII (*NPQ*) (Fig. 8e), and the quantum yield of non-regulated, non-photochemical energy loss in PSII (*NO*) (Fig. 8f) were quite similar in L693 and L661. The value of *ΦPSII* recorded in L693 was significantly lower at the heading stage and significantly greater at 30 days after heading, compared with that in L661 (Fig. 8d). In L693, the value of *NPQ* obtained was significantly greater than that in L661 at the heading stage (Fig. 8e), and the value of *NO* was significantly lower than that in L661 at 30 days after heading (Fig. 8f).

Yields were compared between the various *WCBP1* genotypes and between the resistant plants and the susceptible plants in the F_2_ population (Table 2). L693 exhibited a significantly greater 1000-grain weight (*S*_*w*_) and total grain weight per ear (*T*_*w*_) than that of L661 (*P* <0.05). The number of grains per ear (*N*_*s*_) was also greater (*P* = 0.054). A total of 471 plants from the genetic mapping population was analyzed to determine yields (Additional file 2: Table S5), and resistant plants displayed significantly higher *S*_*w*_, *N*_*s*_ and *T*_*w*_ values compared to those of susceptible plants (Table 2). In addition, homozygous resistant and heterozygous *WCBP1* genotypes showed significantly higher *S*_*w*_, *N*_*s*_ and *T*_*w*_ values than those of homozygous susceptible *WCBP1* genotypes.

**Table 2:** Comparison of the main yield parameters, including the number of grains per ear (*N*_*s*_), total grain weight per ear (*T*_*w*_) and 1000-grain weight (*S*_*w*_), between the L693 and L661 parents and among the different genotypes in the segregating F_2_ population by independent sample t-tests

Yield component	Parent						F_2_						
	Phenotype	Genotype	No. of individuals	Mean	Std. error	*P*-value	Phenotype	Genotype	No. of individuals	Mean	Std. error	P1-value	P2-value
*Ns*	R	L693 (*+WCBP1*/+WCBP1)	15	59.167	4.121	0.054	R	*+WCBP1/+WCBP1*	237	33.810	1.344	0.000**	0.149
							R	*+WCBP1/-WCBP1*	115	32.699	0.852	0.000**	
	S	L661 (+WCBP1/+WCBP1)	12	45.267	5.195		S	*-WCBP1/-WCBP1*	119	22.684	0.983		
*Tw*	R	L693 (*+WCBP1*/+WCBP1)	15	2.020	0.222	0.003**	R	*+WCBP1/+WCBP1*	237	0.841	0.046	0.000**	0.449
							R	*+WCBP1/-WCBP1*	115	0.766	0.032	0.000**	
	S	L661 (+WCBP1/+WCBP1)	12	0.966	0.219		S	*-WCBP1/-WCBP1*	119	0.463	0.031		
*Sw*	R	L693 (*+WCBP1*/+WCBP1)	15	33.338	2.352	0.021*	R	*+WCBP1/+WCBP1*	237	25.537	0.944	0.001**	0.055
							R	*+WCBP1/-WCBP1*	115	21.019	0.492	0.042*	
	S	L661 (+*WCBP1*/+*WCBP1*)	12	20.254	4.351		S	*-WCBP1/-WCBP1*	119	18.379	1.618		

R indicates the resistant phenotype, and S indicates the susceptible phenotype. In the different genotypes, *R* indicates the dominant resistance gene, and *r* indicates the recessive, susceptible allele. P, value describing the differences between L693 and L661; P1, value describing the differences between the *RR* and *Rr* genotypes compared to *rr*; P2, value describing the differences between the *RR* and *Rr* genotypes. Asterisks represent significant differences: **P ≦0.01, *P ≦0.05

## Discussion

### A gene encoding a heavy metal copper-transport protein is involved in response to stripe rust infection

In this study, we identified *WCBP1* and *WCBP2*, two genes that encode predicted copper-binding proteins. Genes encoding heavy metal copper-binding proteins have proven interesting in multiple studies. For example, similar genes exhibited significant changes in expression in all of eight lines near-isogenic for stripe rust resistance [21]. In rice, the *Pi21* gene, encoding a putative heavy metal copper-binding domain protein, confers resistance to blast disease [22]. Furthermore, the rice gene *Xa13*, encoding a heavy metal copper-transport protein, confers resistance to the bacterial pathogen *Xanthomonas oryzae* [23]. In humans, *Hah1* encodes a metallochaperone protein belonging to a family of proteins containing metal-binding domains and is related to the Menkes/Wilson disease protein [24]. These findings indicate that a great diversity of copper-binding protein genes can confer resistance to various fungal and bacterial diseases in different species.

qPCR analysis (Fig. 6a) indicated that the transcription of the *WCBP1* susceptible allele was inhibited. In contrast, *WCBP2* is constitutively expressed in both L693 and L661, and its sequence is identical in these lines (Fig. 6b). However, its expression was only activated by inoculation with *Pst* in L693. *WCBP1* co-segregated with resistance in F_2:3_ populations (Additional file 2: Table S5). Expression of both *WCBP1* and *WCBP2* was not upregulated (*P* <0.05) by mock inoculation with water (Fig. 6c and d), indicating that these genes respond specifically during the resistance response to stripe rust.

A gene encoding a blue copper-binding protein was induced by various oxidative stresses in *Arabidopsis* and transgenic tobacco plants [25–27]. Indeed, there is an oxidative burst during the primary hypersensitive response [21], which implies that the oxidative burst potentially resulting from *Pst* attack may induce the expression of *WCBP1*.

*WCBP1* enhances photosynthetic competence without altering the timing of leaf senescence and protects wheat yields against *Pst* infection.

Another interesting category of differentially expressed genes is the leaf senescence-associated genes (SAGs). Previous studies implied that leaf senescence might be involved in the defense against pathogenic fungi [16]. Transcriptome analysis also confirmed that *TaSAG120* [GenBank: JN558557] is involved in the plant defense against *Pst* infection [15]. A total of 37.5 % (42/112) of sequences were identified as belonging to photosynthesis-like and leaf senescence-like pathways (Fig. 1c). Previous studies showed that many pathogenesis-related (PR) genes are induced during leaf senescence in *Arabidopsis*, and the types and numbers of SAGs present within this family vary from species to species and from genotype to genotype in the same species infected with the same fungal pathogen [24, 28, 29]. Fourteen of the SAG-related ESTs (Additional file 2: Table S2) have very high sequence similarity to *TaSAG120* (99.4 %). This finding further suggests that unique transcripts of SAGs are specific to certain *Yr* genotypes.

*YrL693* confers effective resistance at the seedling and adult stages (Fig. 1a and b). The physiological effects of stripe rust on wheat yields were estimated by measuring photosynthetic parameters at the adult stage because yield losses caused by *Pst* infection occur mostly from disease at the adult stage [5]. Previous studies suggested that a leaf senescence-associated pathway might be involved in the response to stripe rust infection [14–16]. Our data (Fig. 7) suggest that *P*_*n*_ was regulated by the photosynthetic apparatus rather than by stomatal factors in L693, indicating that L693 could actively regulate the photosynthetic pathway following infection with *Pst*. This hypothesis was also supported by observed differences in gene expression.

The increase in photosynthetic competence observed in L693 could be due either to an increase in the photochemical efficiency of PSII or an increase in the efficiency of excitation capture by open PSII reaction centers. The latter phenomenon would result from other aspects of photosynthesis, such as photosynthetic electron transport and light utilization, and could therefore be affected by pathogen infection. The onset of leaf senescence generally occurs at 30 days after heading [30]. At this time point, leaves from the resistant wheat line L693 exhibited significantly higher *F*_*v*_/*F*_*m*_, *F*_*v*_^*‘*^/*F*_*m*_^*’*^ and *ΦPSII* values as well as significantly lower *NO* compared with that of L661 leaves, although the changes in *F*_*v*_/*F*_*m*_, *F*_*v*_^*’*^/*F*_*m*_^*’*^, *q*P, *ΦPSII*, and *NPQ* were very similar (Fig. 8), especially at 20–30 days after heading. These findings indicated that the higher photosynthetic competence observed in L693 resulted from a higher photochemical efficiency of PSII, which is an important, actively regulated mechanism involved in adaptation to disease stress. This result showed that the putative candidate stripe rust resistance gene *WCBP1* did not alter the onset or progression of leaf senescence (Fig. 7). The susceptible allele possibly accelerated the progress of leaf senescence within a normal onset period; therefore, the maintenance of a higher photochemical efficiency in PSII is a direct and important factor in protection of yield against *Pst* infection.

### *YrL693* may have originated from a DNA sequence change in the wheat genome

We originally thought that *YrL693* originated from *Th. intermedium* because the pedigree of L693 includes *Th. intermedium* [18, 31]. However, the fine genetic map of *YrL693* shows similar marker order and total length that is consistent with the chromosome map of 1B (Fig. 3). This result indicates that there is likely no alien chromosome segment in *YrL693*. Comparative genomic analysis of *YrL693* (Fig. 4) also demonstrated that an alien chromosome segment is unlikely. Moreover, genes orthologous to *WCBP1* were also identified in *B. distachyon*, rice, and sorghum (Figs. 3 and 4). Additionally, we did not detect *Th. intermedium* sequences in the genomic region of *YrL693* using closely linked markers. Furthermore, the genetic linkage map showed that *YrL693* is located in the centromeric region, whereas it is commonly thought that insertion or translocation of an alien chromosome segment into the centromeric region very rarely occurs in wheat [32]. All available evidence supports the hypothesis that *YrL693* was not transferred from *Th. intermedium*, so we conclude that *YrL693* is a wheat gene.

### *WCBP1* is a candidate for the *YrL693* stripe rust resistance gene

The sequence of the *WCBP1* allele associated with resistance contains a 36 bp deletion compared to the sequence in the susceptible line (Fig. 2 and Additional file 1: Figure S3). This finding suggests that resistance originated with the 36 bp deletion, similar to the event observed for the blast resistance *Pi21* gene in rice [22]. These findings implied that DNA sequence changes, especially deletions, might be important mechanisms for evolution of disease resistance in plants. Previous studies confirmed that the *Yr36* resistance gene encodes a kinase with a putative lipid-binding domain [33], whereas *Yr18* encodes an adenosine triphosphate-binding cassette transporter belonging to a pleiotropic drug resistance subfamily [16]. These findings indicate that different *Yr* genes have diverse and specific functions.

## Conclusions

In this study, we identified the gene *WCBP1*, which may act as a candidate gene for the wheat stripe rust resistance gene *YrL693* based on the following evidences. First, the DNA sequence of *WCBP1* was polymorphic between L693 and L661, as well as between the parents YU25 and MY11, and results from the polymorphic amplification demonstrated polymorphism between the *WCBP1* alleles in all parental pairs. Second, genetic mapping of *YrL693* showed that *WCBP1* co-segregated with *YrL693*. Third, qPCR analysis confirmed that the expression of *WCBP1* differed significantly between resistant and susceptible alleles at 96 h after *Pst*-inoculation. Moreover, the expression of *WCBP1* in resistant/susceptible plants was upregulated only upon *Pst* infection, while it did not change significantly upon mock-inoculation with water. The fact that *WCBP1* was not annotated previously can be explained by its putative divergence in being specific to certain *Yr* genotypes. It is therefore reasonable to hypothesize that *WCBP1* is a candidate gene for *YrL693*, but positive confirmation will come with appropriate transformation experiments.

## Materials and methods

### Plant materials

The isogenic wheat lines L693 with *YrL693* and L661 without *YrL693* were used to examine differential gene expression after the plants were inoculated with *Pst*. Their parents, YU25 and MY11, were employed as controls for the analysis of the candidate resistance gene. *Th. intermedium* was employed for in situ hybridization to check for alien chromatin in YU25 and L693. A set of Chinese Spring (CS) nulli-tetrasomic (NT) and relevant ditelosomic lines were used to identify the chromosomal location of the candidate resistance gene. Genotypes of 523 F_2:3_ families derived from the cross L661/L693 were used to construct the fine genetic map of the candidate resistance gene.

### Gene expression in plants challenged with *Pst*

Seedlings at the three-leaf stage, grown in a greenhouse, were challenged with urediniospores of *Pst* race CYR32. *Pst* inoculation was conducted according to a previously described method [34]. Leaves from L693 and L661 plants were harvested at 24, 48 and 96 h post-inoculation. A mixture of L693 RNA samples containing equal amounts of RNA extracted at each time point was used as the tester. A mixture of L661 RNA collected at the same time points was used as the driver for the construction of the cDNA library. Non-inoculated leaves from L693 and L661 plants at 0 h, as well as leaves mock-inoculated with water, were harvested for use as controls for gene expression analyses. A sample was collected from L661 at 18 days post-inoculation to represent full disease development (Fig. 1a).

### Construction of the SSH library

Total RNA was extracted from L693 and L661 leaves using the RNA extraction reagent TRIzol (Invitrogen, Carlsbad, CA, USA). The Dynabeads Oligo dT_25_ system (Dynal A.S., Oslo, Norway) was then employed to purify mRNA and to construct cDNA libraries produced by RT-PCR. An SSH library was constructed using the PCR-Select cDNA Subtraction Kit (Clontech, Palo Alto, CA, USA) according to a previously described protocol [30, 35]. Positive colonies were sequenced using an ABI Prism 3100 automated sequencer (Perkin Elmer ABD, Santa Clara, CA, USA). The resulting ESTs were analyzed against the GenBank database using BLASTX and BLASTN. The threshold probability for a sequence match was set at 10^−5^.

### Plant growth conditions

All wheat materials were planted in the field at the Yaan Agricultural Research Station of Sichuan Agricultural University (27°17′ N, 120°16′ E) during the 2010–2011 wheat-growing season (total rainfall, approximately 445 mm). Seeds were sown in a clay soil on October 29, 2010. Each experiment was executed as a randomized complete block design with three replicates. Approximately 25 seeds from each line were planted in 2.5 m rows with 25 cm spacing. The susceptible wheat line SY95-71, serving as a vector of the pathogen, was planted in every third row of each population to ensure that all plants had an equal chance of infection. SY95-71 was artificially inoculated with *Pst* race CYR32 at the seedling stage according to a previously described method [36]. The average air temperatures from sowing to grain maturity ranged from 8.8 to 22.5 °C. The heading date of both L693 and L661 was approximately March 15, 2011. The plants received 3, 4, 4 and 8 g N m^−2^ ammonium nitrate at the following growth stages, respectively: one-node, meiosis, heading and anthesis. After planting, 10 % Imidacloprid wettable powder (1-[6-chloro-3-pyridylmethyl]-nitroimidazolidin-2-ylideneamine; Yangnong Chemical Group Company, Jiangsu) was applied to control pests. Thirty plants from each genotype showing identical growth and developmental characteristics at heading were marked for all subsequent measurements and observations.

### Measurement of photosynthetic indices and PSII activity

Photosynthetic parameters and chlorophyll fluorescence were measured in L693 and L661 using a portable photosynthesis system (Li-6400-02B, Li-Cor, USA) with a red-blue light source and a modulated photosynthesis system (Li-Cor 6400XT, Lincoln, NE, USA), respectively. Data collection was carried out once every 10 days, starting from heading on March 15, 2011, through April 14, 2011. At each sampling date, the mean of three independent measurements from each plant represented the plant phenotypic value, and the average values from 10 plants were employed for comparisons between genotypes. Measurements, parameter recording, and parameter calculations followed previously described methods [30, 37, 38].

### In silico mapping of candidate genes

The genomic locations of the wheat ESTs were determined *in silico* via BLASTN searches against the mapped expressed wheat sequences using GrainGenes 2.0 [39] at a 10^−5^ threshold probability and via a BLASTX search against the wheat draft genome [40]. Sequences without a BLASTX hit or those showing an e-value of less than 10^−5^ were excluded from the *in silico* mapping to the wheat genome and the *Brachypodium* genome.

### PCR-based mapping of polymorphic ESTs and linkage analysis for resistance

Primers were designed using Primer3 [41] to amplify ESTs from wheat genomic DNA. Wheat genomic DNA was extracted using a previously described method [42]. Primer sequences were listed in Additional file 2: Table S3. PCR amplification and product analysis were performed according to previously described methods [19, 43].

Polymorphic primers were used to genotype the segregating population of 523 F_2:3_ families (Additional file 2: Table S5). Linkage analysis of markers and resistance genes was performed with JoinMap 4 [44]. The linkage group was declared at a LOD threshold of 3.0. Subsequently, the chromosomal locations of the linked markers were confirmed using Chinese Spring nullisomic-tetrasomic and the ditelosomic lines of chromosome 1B [45].

### PCR-based cloning of putative stripe rust resistance genes

The primers designed with Primer3 (Additional file 2: Table S4 and S6) according to the contig sequence containing the co-segregated markers and the polymorphic amplicon sequence [46] were employed to amplify the entire length of the candidate gene. These primers were listed in Additional file 2: Table S6. Each amplicon was purified using the GeneJET PCR Purification Kit (Thermo Scientific, Beijing) and cloned into the pGEM T Easy Vector (Promega). JM109-competent cells were transformed and cultured according to the manufacturer’s protocol. Plasmids retrieved using the GeneJET Plasmid Miniprep Kit (Thermo Scientific) were sequenced by Shanghai Majorbio Bio-Pharm Technology Co., Ltd. (Shanghai, China).

### qRT-PCR of the candidate gene and the key unigene conferring resistance

We used M-MLV (Invitrogen) to synthesize complementary DNA (cDNA) for real-time qRT-PCR (Invitrogen). Each 20 μl reaction mixture contained 4 μg of RNA. For qRT-PCR assays, 2 μl of the diluted cDNA (1:20) and 9 μl of RealMasterMix (SYBR Green) (TIAGEN) were included in 20 μl reaction volumes. qRT-PCR was conducted using the MiniOpticon Time PCR Detection System with the following program: pre-incubation at 95 °C for 5 min, followed by amplification for 40 cycles at 95 °C for 30 s and 62 °C for 30 s. The melting curve was set at 65 to 95 °C, with a 0.5 °C increase per step, and cooling was set at 40 °C for 30 s. Each qRT-PCR run was replicated with three independent biological samples, and three technical repeats were included for each biological sample. The primer sequences and descriptions of the genes are provided in Additional file 2: Table S7. The crossing point value and results of the melting curve analyses were obtained using Roche CFX Manager Software for the MiniOpticon System 3.0 (Hercules, CA, USA). Melting curve data showing only a single peak, which denotes primer specificity (Additional file 1: Figure S1a), were collected for all samples. The repeatability and reliability of amplifications are demonstrated by the raw data shown in Additional file 1: Figure S1b.

### Statistical analysis

Significant differences in the mean physiological parameters, gene expression, and yield between L693 and L661 and between plants carrying and lacking WCBP1 were observed. Differences between two adjacent time points for the same genotype were determined using independent sample t-tests in IBM SPSS Statistics 19 (SPSS Inc., Chicago, IL, USA).

## Additional files

Additional file 1: Figure S1. Length distributions of the wheat leaf ESTs (A) and the assembled uniESTs (B), including contigs (C) and singletons (D). **Figure S2.** Primers and the amplified region of genomic DNA. To clone the *WCBP1* gene, primers were designed according to the Chinese Spring draft genome sequence. Full-length amplification was performed using the 1B-1 forward primer and the 1B-6 reverse primer. **Figure S3.** Comparison of *WCBP1* gene sequences. *WCBP1* encodes a heavy metal copper-binding protein with two copper sensing domains. The four sequences were cloned using PCR with primers designed according to the polymorphic fragment. L693 is the stripe rust-resistant line; L661 is the stripe rust-susceptible line; YU25 is the resistant parent of L693 and L661; MY11 is the susceptible parent of L693 and L661. **Figure S4.** Quality map of the sequences flanking the SNP site. A represents L693; B represents YU25; C represents L661; D represents MY11, which showed that the SNP existed among the different genotypes. **Figure S5.** GISH analysis of mitotic metaphase chromosomes of TAI 7047, L661, L693 and YU25 using genomic DNA of Th. Intermedium as a probe (green). A TAI 7047, B L661, C L693, D YU25. Chromosomes were counterstained with DAPI (blue). **Figure S6.** Results demonstrating the high quality and good replication of q-PCR. (A), the melt curve of the q-PCR product during the amplification of *WCBP2*. (B), The amplification curve shows the consistent crossing points of the three technological replicates of the *WCBP2* gene and the reference gene *GAPDH*. This chart shows L661 at 0 h. (ZIP 837 kb) Additional file 2: Table S1. . Detailed information on 112 ESTs. A BLAST search was performed using BLASTX in NCBI. In silico mapping was performed using the IWGSC draft wheat sequence database. **Table S2.** BLAST searches performed for the forward subtraction clones found in the stripe rust-resistant genotype L693. **Table S3.** The primers used to amplify ESTs. Primer pairs were only designed for 62 out of the 112 ESTs because the remaining ESTs were too short to be used for primer design. **Table S4.** Primers were designed according to the sequence of the contig (3837062) carrying the polymorphic amplicon, which was located on wheat chromosome 1B (http://www.wheatgenome.org/). These amplicons were used to determine the linkage relationship between the markers and the stripe rust resistance gene in L693. **Table S5.** Results of the amplification of 5 WCBP1-linked genetic markers in an F_2_ population derived from crosses between L661 and L693 and between L693 and L661. The genotypes (RR, Rr, rr) of the F_2_ individuals were determined according to the phenotypes of the corresponding F_2:3_ families; "A" and "B" for the individual F_2_ populations were derived from crosses between L661 and L693 and between L693 and L661, respectively. R represents the same amplicon as the resistant parent L693; S represents the same amplicon as the susceptible parent L661; H represents the heterozygous condition. The yield components of the F_2_ plants, including the number of grains per ear (*N*_*s*_), the total grain weight per ear (*T*_*w*_) and the 1000-grain weight (*S*_*w*_), are provided. ‘\’ represents a missing yield component in the F_2_ plants. **Table S6.** The sequences of seven primer pairs designed according to the sequence of a cloned candidate gene in silico; these primers were used for PCR-based gene cloning from genomic DNA. **Table S7.** Primers for q-PCR analysis. (ZIP 94 kb) Additional file 3:
**Methods S1.** Genomic in situ hybridization (GISH). (PDF 13 kb)

## Abbreviations

bp: Base pair
ESTs: Expressed sequence tags
ORF: Open reading frame
PR: Pathogenesis-related
*Pst*: *Puccinia striiformis* f. sp. *tritici*
qRT-PCR: Quantitative reverse transcription PCR
SAG: Senescence-associated gene
SNP: Single nucleotide polymorphism
SSH: Suppression subtractive hybridization
SSR: Simple sequence repeat
WCBP: Wheat copper-binding protein
