# Supplementary material for: Wheat WCBP1 encodes a putative copper-binding protein involved in stripe rust resistance and inhibition of leaf senescence
Source: BMC Plant Biol. 2015 Oct 6;15:239. doi: 10.1186/s12870-015-0612-4 (PMC4595213; doi:10.1186/s12870-015-0612-4)

Figure S3. Comparison of *WCBP1* gene sequences. *WCBP1* encodes a heavy metal copper-binding protein with two copper sensing domains. The four sequences were cloned using PCR with primers designed according to the polymorphic fragment. L693 is the stripe rust-resistant line; L661 is the stripe rust-susceptible line; YU25 is the resistant parent of L693 and L661; MY11 is the susceptible parent of L693 and L661.

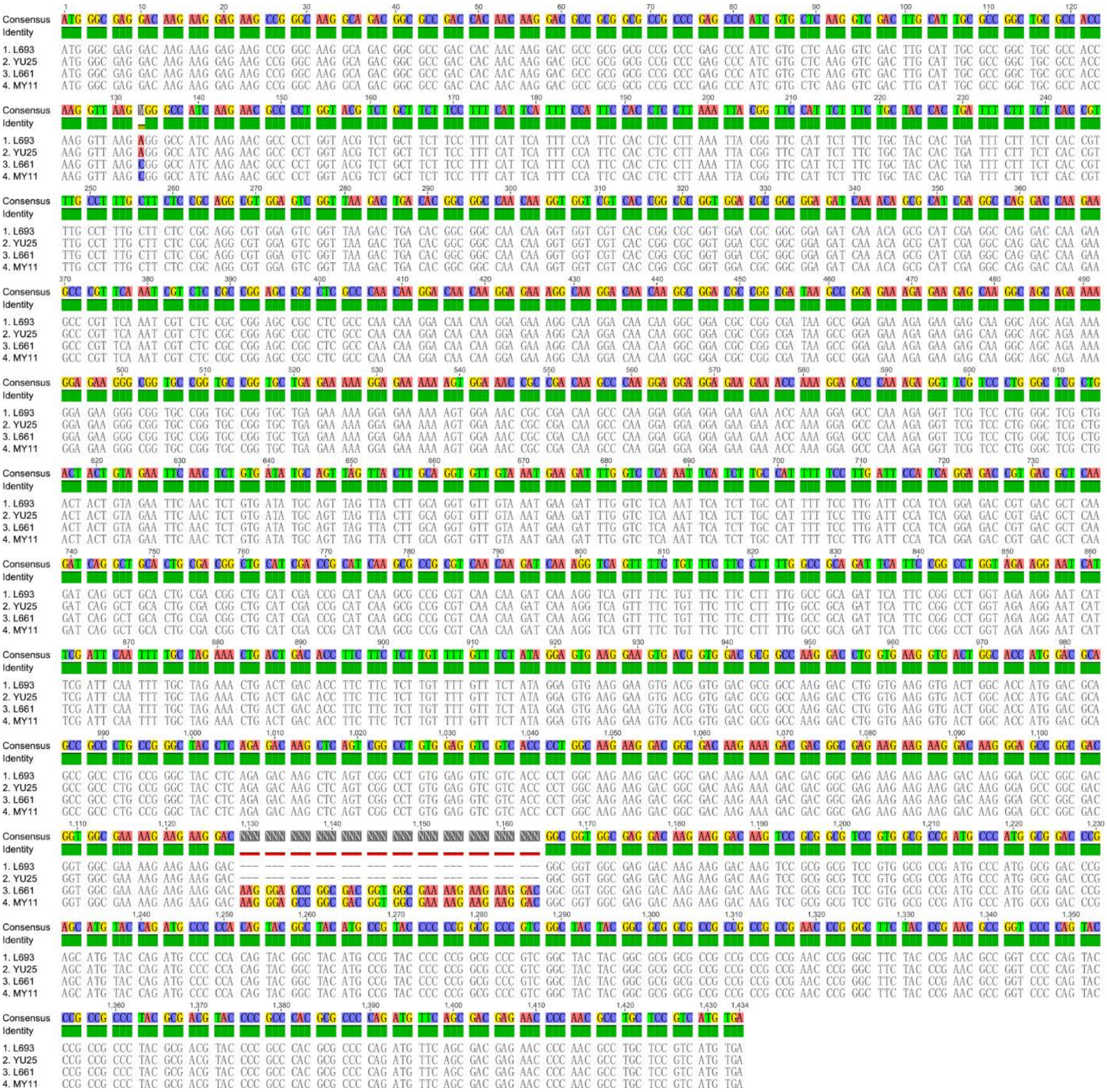

Supplement: Additional file 1: Figure S1. — Length distributions of the wheat leaf ESTs (A) and the assembled uniESTs (B), including contigs (C) and singletons (D). Figure S2. Primers and the amplified region of genomic DNA. To clone the WCBP1 gene, primers were designed according to the Chinese Spring draft genome sequence. Full-length amplification was performed using the 1B-1 forward primer and the 1B-6 reverse primer. Figure S3. Comparison of WCBP1 gene sequences. WCBP1 encodes a heavy metal copper-binding protein with two copper sensing domains. The four sequences were cloned using PCR with primers designed according to the polymorphic fragment. L693 is the stripe rust-resistant line; L661 is the stripe rust-susceptible line; YU25 is the resistant parent of L693 and L661; MY11 is the susceptible parent of L693 and L661. Figure S4. Quality map of the sequences flanking the SNP site. A represents L693; B represents YU25; C represents L661; D represents MY11, which showed that the SNP existed among the different genotypes. Figure S5. GISH analysis of mitotic metaphase chromosomes of TAI 7047, L661, L693 and YU25 using genomic DNA of Th. Intermedium as a probe (green). A TAI 7047, B L661, C L693, D YU25. Chromosomes were counterstained with DAPI (blue). Figure S6. Results demonstrating the high quality and good replication of q-PCR. (A), the melt curve of the q-PCR product during the amplification of WCBP2. (B), The amplification curve shows the consistent crossing points of the three technological replicates of the WCBP2 gene and the reference gene GAPDH. This chart shows L661 at 0 h. (ZIP 837 kb) [file 12870_2015_612_MOESM1_ESM.zip › Additional file 3.pdf]
