# Supplementary material for: Wheat WCBP1 encodes a putative copper-binding protein involved in stripe rust resistance and inhibition of leaf senescence
Source: BMC Plant Biol. 2015 Oct 6;15:239. doi: 10.1186/s12870-015-0612-4 (PMC4595213; doi:10.1186/s12870-015-0612-4)
Supplement: Additional file 3: — Methods S1. Genomic in situ hybridization (GISH). (PDF 13 kb) [file 12870_2015_612_MOESM3_ESM.pdf]

Methods S1

Genomic *in situ* hybridization (GISH)

Seeds were germinated on moistened filter paper in petri dishes. Actively growing roots were removed from seedlings and placed in gas treatment for 2 h, fixed in 90% acetic acid and stored in 70% v/v ethanol. Chromosome spread preparation was performed as previously described (Han et al., 2006). Genomic DNA of Th. elongatum was isolated by a modified CTAB method (Kidwell and Osborn, 1992) and labeled with f Chroma Tide Alexa Fluor 488-5- dUTP (Invitrogen) by the nick translation method and used as a probe. The probe was blocked with genomic DNA of "Chinese Spring". Detection and visualization were performed as described (Han et al., 2009).

Supplemental References

Han, F.P., Lamb, J.C., and Birchler, J.A. (2006) High frequency of centromere inactivation resulting in stable dicentric chromosomes of maize. *Proc. Natl. Acad. Sci. USA*, **103**, 3238–3243.

Han, F.P., Gao, Z., and Birchler, J.A. (2009) Centromere inactivation and reactivation reveal both genetic and epigenetic components for centromere specification. *Plant Cell*, **21**, 1929–1939.

Kidwell, K.K., and Osborn, T.C. (1992) Simple plant DNA isolation procedures. In JS Beckmann, TC Osborn, eds. Methods for Genetic and Physical Mapping, Kluwer, Dordrecht, pp 1–13.
